# Pneumatic Versus Holmium:YAG Laser Lithotripsy During True Tubeless Mini-Percutaneous Nephrolithotomy: A Retrospective Comparative Study

**DOI:** 10.7759/cureus.113818

**Published:** 2026-08-02

**Authors:** Jorge A Martin Millet, David J Saavedra Verduga, Katia J Bracamontes Gil, Josselyn Martinez Conce, Eduardo Cruz Nuricumbo

**Affiliations:** 1 Department of Urology, Hospital Regional de Alta Especialidad de la Peninsula de Yucatan (HRAEPY), Merida, MEX; 2 Department of Endourology, Hospital Regional de Alta Especialidad de la Peninsula de Yucatan (HRAEPY), Merida, MEX

**Keywords:** holmium-yag laser lithotripsy, mini-percutaneous nephrolithotomy (mini-pcnl), nephrolithiasis, percutaneous nephrolithotomy (pcnl), pneumatic lithotripsy, renal calculi (kidney stones), stone-free rate (sfr), tubeless pcnl, urolithiasis

## Abstract

Purpose: The purpose of this study is to compare stone-free and safety outcomes between pneumatic and Holmium:YAG laser lithotripsy in patients undergoing true tubeless mini-percutaneous nephrolithotomy (mini-PCNL) for renal calculi.

Materials and methods: A retrospective comparative study was conducted including 34 consecutive adult patients who underwent true tubeless mini-PCNL between 2021 and 2024 at a tertiary referral center. Procedures were performed using a standardized 16-Fr tract with an 11/12-Fr operating sheath. Stone fragmentation was achieved using either pneumatic lithotripsy (Swiss LithoClast®) or a 100-W Holmium:YAG laser with a 200-µm fiber. The lithotripsy modality was selected according to surgeon preference, equipment availability, and intraoperative considerations. Stone-free status was defined as the absence of residual fragments or residual fragments <3 mm on non-contrast computed tomography performed at least 30 days after surgery. The primary outcome was the CT-confirmed stone-free rate. Secondary outcomes included estimated blood loss, postoperative sepsis, and 30-day complications.

Results: Thirty-four patients were included, with 17 patients in each group. Baseline characteristics were comparable. Endoscopic stone-free rates were achieved in 15 (88.2%) and 14 (82.4%) patients in the pneumatic and Holmium:YAG laser groups, respectively (p=0.628). CT-confirmed stone-free rates were achieved in 12 (70.6%) and nine (52.9%) patients, respectively (p=0.290). Estimated blood loss and postoperative complications were comparable between groups. One patient (5.9%) in the Holmium:YAG laser group developed a Clavien-Dindo grade IV postoperative hemorrhagic complication requiring blood transfusion. Postoperative sepsis occurred in 0 (0.0%) patients.

Conclusions: In this homogeneous cohort of patients undergoing true tubeless mini-PCNL, Holmium:YAG laser lithotripsy did not demonstrate superior stone-free or safety outcomes compared with pneumatic lithotripsy. Both modalities achieved comparable clinical outcomes and represent effective treatment options for appropriately selected patients.

## Introduction

Renal stone disease is a common urological condition and represents an increasing global health burden. Its management depends on stone size, location, composition, anatomical factors, and patient-related characteristics. Current treatment options include extracorporeal shock wave lithotripsy, retrograde intrarenal surgery, and percutaneous nephrolithotomy, with percutaneous nephrolithotomy remaining the standard approach for larger or complex renal stones [1,2].

Mini-percutaneous nephrolithotomy (mini-PCNL) was developed to reduce the morbidity associated with standard percutaneous access while preserving adequate stone clearance. Recent evidence on miniaturized PCNL has focused on perioperative risk prediction, factors affecting stone clearance, and the evolution of miniaturized and ultra-mini techniques [3-5]. The miniaturization of percutaneous instruments may decrease blood loss, postoperative pain, and hospital stay, although stone-free outcomes remain influenced by stone burden, stone density, calyceal anatomy, access strategy, and the lithotripsy device used.

Several intracorporeal lithotripsy technologies are available during mini-PCNL. Pneumatic lithotripsy remains widely used because of its effectiveness, availability, and relatively low cost, including in miniaturized percutaneous approaches [6]. Comparative evidence across intracorporeal lithotripsy devices and clinical series using pneumatic or holmium laser lithotripsy supports the relevance of evaluating these modalities in contemporary endourological practice [7-9]. However, pneumatic lithotripsy may be associated with stone migration and the need for fragment retrieval. Laser lithotripsy, particularly holmium laser technology, allows controlled fragmentation and dusting with potentially lower retropulsion. Nevertheless, comparative evidence in mini-PCNL, including multicenter and prospective comparative studies, remains mixed regarding whether laser-based fragmentation provides superior stone-free or safety outcomes compared with mechanical or pneumatic lithotripsy [10-12].

Therefore, given the limited evidence from standardized true tubeless mini-PCNL procedures and the inconsistent findings regarding the comparative effectiveness of intracorporeal lithotripsy modalities, this study aimed to compare stone-free outcomes and perioperative safety between pneumatic and Holmium:YAG laser lithotripsy in adult patients undergoing mini-PCNL at a tertiary referral center in Mexico.

## Materials and methods

Study design and setting

We conducted a single-center, retrospective, comparative cohort study at the Department of Urology, Hospital Regional de Alta Especialidad de la Península de Yucatán, Mérida, Yucatán, Mexico. Medical records of adult patients who underwent mini-PCNL between 2021 and 2024 were reviewed.

Patient selection

Patients aged 18 years or older who underwent mini-PCNL with either pneumatic or laser lithotripsy were included. Patients were excluded if they were younger than 18 years, underwent standard or ultra-mini percutaneous nephrolithotomy, received combined pneumatic and laser lithotripsy during the same procedure, underwent bilateral procedures, had incomplete medical records, lacked postoperative computed tomography for assessment of stone-free status, or were lost to follow-up. Included patients were required to have complete clinical records, including documentation of stone-free status, estimated blood loss, postoperative sepsis, and complications classified according to the Clavien-Dindo system [13]. All consecutive eligible patients who met the inclusion criteria and none of the exclusion criteria were included in the final analysis.

Patients were assigned to two groups according to the lithotripsy modality documented in the operative report: pneumatic lithotripsy and laser lithotripsy. All eligible cases during the study period were included; therefore, no formal sample size calculation was performed.

Surgical technique

All procedures were performed using a standardized mini-PCNL technique in the modified supine position under fluoroscopic guidance. A single percutaneous renal access tract was established in all patients and dilated to 16 Fr using an 11/12-Fr operating sheath. Stone fragmentation was performed using either a pneumatic lithotripter (Swiss LithoClast®, EMS, Switzerland) or a 100-W Holmium:YAG laser (Lumenis Ltd., Yokneam, Israel) with a 200-µm laser fiber, according to the lithotripsy modality documented in the operative report. Laser settings were selected at the discretion of the operating surgeon. Continuous irrigation was provided by gravity throughout the procedure.

True tubeless mini-PCNL was defined as completion of the procedure without placement of either a nephrostomy tube or a double-J ureteral stent. Stone fragments were extracted as required until satisfactory endoscopic stone clearance was achieved.

Variables and outcomes

The following variables were collected: age, sex, stone burden, stone density on computed tomography, estimated blood loss, endoscopic stone-free status, CT-confirmed stone-free status during follow-up, postoperative sepsis, and postoperative complications.

Stone density was recorded in Hounsfield units. Stone burden was expressed in mm². Stone-free status was defined as the absence of residual fragments on endoscopic assessment or follow-up computed tomography, according to the assessment modality. CT-confirmed stone-free status was assessed at a minimum follow-up of 30 days. Postoperative complications were evaluated within 30 days after surgery and graded according to the Clavien-Dindo classification.

The primary outcome was stone-free status after mini-PCNL. Secondary outcomes included estimated blood loss, postoperative sepsis, and 30-day postoperative complications.

Statistical analysis

Statistical analysis was performed using IBM SPSS Statistics for Windows, Version 24 (Released 2016; IBM Corp., Armonk, New York, United States). The Kolmogorov-Smirnov test was used to assess the distribution of continuous variables. Continuous variables were compared using Student’s t-test or the Mann-Whitney U test, according to data distribution. Categorical variables were compared using the chi-square test or Fisher’s exact test, as appropriate. A two-sided p-value <0.05 was considered statistically significant.

Ethics

The study was approved by the Institutional Ethics Committee of the Hospital Regional de Alta Especialidad de la Península de Yucatán on November 14, 2024, under approval code 2024-012. Because of the retrospective nature of the study and the use of anonymized clinical records, the requirement for informed consent was waived. The study was conducted in accordance with the principles of the Declaration of Helsinki.

## Results

A total of 34 patients who underwent mini-PCNL between 2021 and 2024 were included. Patients were evenly distributed between the pneumatic lithotripsy group and the laser lithotripsy group, with 17 patients in each group. The mean age of the overall cohort was 46.6 ± 10.6 years. Sex distribution was identical between groups, with 12 (70.6%) female patients in each cohort.

Regarding baseline characteristics, median stone burden was 217.9 mm² in the pneumatic lithotripsy group and 282.6 mm² in the laser lithotripsy group, with no statistically significant difference between groups (p=0.205). Mean stone density was 1016.6 ± 443.7 HU in the pneumatic group and 1223.9 ± 442.8 HU in the laser group (p=0.182). Estimated blood loss was similar between groups, with a median of 100.0 mL in both cohorts (p=0.118) (Table 1).

**Table 1:** Baseline Characteristics of Patients According to Lithotripsy Modality Values are presented as median (interquartile range), mean ± standard deviation, or number (percentage), as appropriate. HU: Hounsfield units; IQR: interquartile range; SD: standard deviation; mini-PCNL: mini-percutaneous nephrolithotomy.

Characteristic	Pneumatic mini-PCNL (n=17)	Laser mini-PCNL (n=17)	p-value
Female sex, n (%)	12 (70.6)	12 (70.6)	1
Male sex, n (%)	5 (29.4)	5 (29.4)	1
Stone burden, mm², median (IQR)	217.9 (140.1–360.9)	282.6 (208.7–507.5)	0.205
Stone density, HU, mean ± SD	1016.6 ± 443.7	1223.9 ± 442.8	0.182
Estimated blood loss, mL, median (IQR)	100.0 (17.5–137.5)	100.0 (100.0–200.0)	0.118

Endoscopic stone-free status was achieved in 15 (88.2%) patients in the pneumatic lithotripsy group and 14 (82.4%) patients in the laser lithotripsy group. This difference was not statistically significant (p=0.628). On follow-up CT, stone-free status was confirmed in 12 (70.6%) patients in the pneumatic group and nine (52.9%) patients in the laser group, without a statistically significant difference between groups (p=0.290).

Postoperative complications within 30 days were uncommon. One patient (5.9%) in the laser lithotripsy group developed postoperative bleeding requiring blood transfusion, classified as Clavien-Dindo grade II. Postoperative complications occurred in 0 (0.0%) patients in the pneumatic lithotripsy group, and postoperative sepsis occurred in 0 (0.0%) patients in both groups (Table 2).

**Table 2:** Stone-Free and Safety Outcomes According to Lithotripsy Modality Stone-free status was assessed endoscopically at the end of surgery and by non-contrast computed tomography at a minimum follow-up of 30 days. The only postoperative complication was postoperative bleeding in one patient in the laser mini-PCNL group, classified as Clavien-Dindo grade II. CT: computed tomography; mini-PCNL: mini-percutaneous nephrolithotomy.

Outcome	Pneumatic mini-PCNL (n=17)	Laser mini-PCNL (n=17)	p-value
Endoscopic stone-free status, n (%)	15 (88.2)	14 (82.4)	0.628
CT-confirmed stone-free status, n (%)	12 (70.6)	9 (52.9)	0.29
30-day postoperative complications, n (%)	0 (0.0)	1 (5.9)	0.31
Clavien-Dindo grade II complication, n (%)	0 (0.0)	1 (5.9)	0.31
Postoperative sepsis, n (%)	0 (0.0)	0 (0.0)	—

## Discussion

In this retrospective comparative study, pneumatic and laser lithotripsy during mini-PCNL showed comparable stone-free and safety outcomes. Although the pneumatic lithotripsy group demonstrated numerically higher endoscopic stone-free rates (15 (88.2%) vs. 14 (82.4%)) and CT-confirmed stone-free rates (12 (70.6%) vs. 9 (52.9%)), these differences did not reach statistical significance. Estimated blood loss was similar between groups, and postoperative complications were infrequent, with only one Clavien-Dindo grade II bleeding complication observed in the laser lithotripsy cohort.

The choice of intracorporeal lithotripsy device during mini-PCNL remains clinically relevant. Pneumatic lithotripsy is widely available, effective, and cost-efficient, making it particularly useful in resource-conscious settings. Its main limitation is the potential for fragment migration, which may require additional retrieval maneuvers. In contrast, laser lithotripsy allows controlled fragmentation and dusting, with potentially reduced retropulsion in selected scenarios. However, the theoretical advantages of laser lithotripsy do not always translate into superior stone-free outcomes, especially when stone burden, stone density, access strategy, calyceal anatomy, and surgeon experience are taken into account [10,11].

Our findings are consistent with previous comparative studies and contemporary evidence suggesting that both pneumatic and laser lithotripsy can achieve satisfactory stone-free rates during mini-PCNL [10-12]. Cormio et al. reported contemporary multicenter data comparing laser versus mechanical lithotripsy in suction mini-PCNL [10], Manzo et al. evaluated Trilogy versus high-power Ho:YAG laser lithotripsy in mini-PCNL [11], and Sharma et al. reported similar stone-free and postoperative complication outcomes when comparing pneumatic and laser lithotripsy in mini-PCNL [12]. In the present cohort, endoscopic stone-free status was achieved in 15 (88.2%) patients in the pneumatic lithotripsy group and 14 (82.4%) patients in the laser lithotripsy group, whereas CT-confirmed stone-free status was achieved in 12 (70.6%) and nine (52.9%) patients, respectively. As expected, CT-confirmed stone-free rates were lower because of the greater sensitivity of computed tomography for detecting residual fragments. This difference highlights the importance of clearly defining the imaging modality and timing used to assess stone-free status in studies evaluating endourological outcomes.

The numerically higher CT-confirmed stone-free rate (12 (70.6%) vs. 9 (52.9%)) observed in the pneumatic group should be interpreted cautiously. This finding may reflect differences in stone characteristics, access tract efficiency, fragmentation strategy, or surgeon preference rather than a true superiority of pneumatic lithotripsy. Moreover, the small sample size limits the statistical power to detect clinically meaningful differences between groups. Therefore, these results should be considered hypothesis-generating rather than definitive.

The observed safety profile was consistent with the expected morbidity of miniaturized percutaneous techniques. Only one postoperative complication occurred in one (5.9%) patient, postoperative bleeding requiring blood transfusion (Clavien-Dindo grade II) in the laser group, whereas postoperative sepsis occurred in 0 (0.0%) patients. Miniaturized percutaneous techniques were developed to reduce morbidity associated with standard percutaneous nephrolithotomy, particularly bleeding and postoperative recovery. In this context, our findings support the feasibility and safety of both lithotripsy modalities when performed in appropriately selected patients. Contemporary evidence on bleeding risk and tubeless mini-PCNL further supports the acceptable safety profile of miniaturized percutaneous approaches in selected patients [14,15].

This study has several limitations. Its retrospective design and single-center setting introduce the possibility of selection bias and limit the generalizability of the findings. The small sample size reduces statistical power and precludes robust subgroup analyses according to stone location, composition, access characteristics, or surgeon experience. Furthermore, operative time, fluoroscopy time, and several procedural variables were not consistently available because of the retrospective nature of the study and therefore could not be included in the analysis. In addition, the lack of randomization means that the choice of lithotripsy modality may have been influenced by clinical or technical factors not fully captured in the medical records. Variability in surgical technique, access strategy, and follow-up imaging timing may also have influenced the observed outcomes. Therefore, these results should be interpreted as hypothesis-generating and should be confirmed in larger prospective studies.

## Conclusions

In this retrospective cohort, pneumatic and laser lithotripsy during mini-PCNL showed comparable stone-free rates and safety outcomes. Although stone-free rates were numerically higher in the pneumatic lithotripsy group, no statistically significant differences were observed between groups. Both modalities may be considered feasible and safe options in appropriately selected patients. Larger prospective studies are required to confirm these findings and better define the role of each lithotripsy modality during mini-PCNL.
